# Antimicrobial use trends, Israel, 2012 to 2017

**DOI:** 10.2807/1560-7917.ES.2019.24.34.1900022

**Published:** 2019-08-22

**Authors:** Yaakov Dickstein, Elizabeth Temkin, Debby Ben-David, Yehuda Carmeli, Mitchell J Schwaber

**Affiliations:** 1 National Center for Infection Control, Ministry of Health, Tel Aviv, Israel; 2 Sackler Faculty of Medicine, Tel Aviv University, Tel Aviv, Israel

**Keywords:** antibiotic, antimicrobial use, Israel, post-acute care hospital, stewardship, surveillance

## Abstract

**Background:**

In 2012, Israel’s National Center for Infection Control initiated a national stewardship programme that included mandatory annual reporting of antimicrobial use. Here we present nationwide Israeli data for the period 2012 to 2017.

**Aim:**

The goal of this study was to detect trends in antimicrobial use in Israel following the introduction of the stewardship programme, as part of an assessment of the programme’s impact.

**Methods:**

In this retrospective observational study, data were collected from Israel’s health maintenance organisations (HMOs), acute care hospitals and post-acute care hospitals (PACHs). Acute care hospital data were collected for general medical and surgical wards, and medical/surgical intensive care units (ICUs). Data were converted into defined daily doses (DDD), with use rates presented as DDD per 1,000 insured/day in the community and DDD per 100 patient-days in hospitals and PACHs. Trends were analysed using linear regression.

**Results:**

Antimicrobial use decreased across sectors between 2012 and 2017. In the community, the decrease was modest, from 22.8 to 21.8 DDD per 1,000 insured per day (4.4%, p = 0.004). In acute care hospitals, antibiotic DDDs per 100 patient-days decreased from 100.0 to 84.0 (16.0%, p = 0.002) in medical wards, from 112.8 to 94.2 (16.5%, p = 0.004) in surgical wards and from 154.4 to 137.2 (11.1%, p = 0.04) in ICUs. Antimicrobial use decreased most markedly in PACHs, from 29.1 to 18.1 DDD per 100 patient-days (37.8%, p = 0.005).

**Conclusion:**

Between 2012 and 2017, antimicrobial use decreased significantly in all types of healthcare institutions in Israel, following the introduction of the nationwide antimicrobial stewardship programme.

## Background

Considerable regional and temporal variation exists in antimicrobial use around the world [1,2]. Reasons for these differences include differing healthcare structures and policies, socioeconomic factors and cultural values [3]. As increasing rates of antimicrobial use in the community and healthcare institutions are associated with the rise of bacteria resistant to antibiotics, it is not surprising that the prevalence of antibiotic-resistant bacteria also varies considerably around the world [4-8]. Antimicrobial stewardship (AST) aimed at curbing antibiotic-resistant bacteria may include a variety of interventions such as audits, antimicrobial use guidelines for common infectious disease syndromes and rapid diagnostic tests, and is dependent on antimicrobial use monitoring to identify and correct problematic prescribing and usage habits [9]. Properly implemented AST has been shown to decrease mortality risk for individual patients [10], in addition to having beneficial effects on public health and limiting the spread of multidrug-resistant organisms [11,12].

To date, relatively little data have been published on antimicrobial use in Israel. An article from the country’s largest health maintenance organisation (HMO), which insures slightly more than 50% of the population, summarised antimicrobial use data in the community [13]. The authors found that between 2000 and 2010, the rate of consumption remained steady at 23.2 defined daily doses (DDD) per 1,000 insured. Four previous articles examined antimicrobial use within Israeli hospitals, three for the period 1998 to 2004 [14-17]. The largest of the studies summarised data from 26 medical wards in six hospitals between 2003 and 2004 and found an overall rate of antimicrobial use of 79.9 DDD per 100 patient-days [17], with considerable variation between wards. An article summarising data from a single Israeli hospital in 1998 found rates, expressed in DDD per 100 bed-days, of 232, 173 and 149 for intensive care units (ICUs), medical and surgical wards, respectively [16].

Restrictive antimicrobial use and infectious disease (ID) approval for certain broad-spectrum agents were common practices in most acute care hospitals for many years before the formation of the Israeli National Center for Infection Control (NCIC), a branch of the Ministry of Health (MOH) [14]. However, after the NCIC was formed, it identified nationally monitored antimicrobial stewardship as an important component of the strategy to confront antimicrobial resistance. In 2012, the NCIC instituted a nationwide programme for judicious use of antimicrobials [18]. As part of the programme, a circular was released requiring all healthcare institutions—including HMOs, acute care hospitals and post-acute care hospitals (PACHs)—to establish an antimicrobial stewardship committee that advises the institution’s director on measures recommended for the judicious use of antimicrobials. While the creation of institutional antimicrobial use guidelines is mandatory, the nature and implementation of specific interventions to reduce antimicrobial use are at the institutions’ discretion. Institutions must submit annual reports to the MOH on antimicrobial use. These are analysed, and annual comparative reports on antimicrobial use are prepared by the MOH and subsequently distributed to the institutions to support antimicrobial stewardship efforts.

The aim of this report is to present antimicrobial use trends in Israel for the period 2012 to 2017, following the launch of the programme, and to compare our data with similar data from Europe.

## Methods

### Study design

This report is an observational, retrospective analysis of aggregate data on antimicrobial use. Antimicrobial dispensing data were collected in the community from all clinics operating under the auspices of the four nationwide HMOs, which cover 100% of the population, and from acute care hospitals and PACHs. The HMOs represent all insurers within the national healthcare programme. From acute care hospitals, data were gathered for medical wards, general surgical wards and medical/surgical intensive care units (ICUs). The PACHs include patient populations requiring sub-acute medical care, inpatient rehabilitation, chronic mechanical ventilation and those fully dependent on nursing care for their activities of daily living.

### Data sources

HMOs reported antimicrobials prescribed to individuals. Hospitals and PACHs used pharmacy databases to report antimicrobials dispensed to wards. Each institution used its pharmacy-operated system to generate the data, which were extracted to Microsoft Excel spreadsheets and submitted for analysis. Data on verified patient consumption of antimicrobials, such as from electronic medical records documenting drug administration, were not accessible. For the sake of simplicity, we refer to antimicrobial dispensing as antimicrobial use or consumption. Data were converted into DDD and grouped into categories using the World Health Organization (WHO) Anatomical Therapeutic Chemical (ATC) classification method (2016 definitions) [19,20]. Use rates from HMO prescriptions were presented as DDD per 1,000 insured per day. Rates for hospitals and PACHs were presented as DDD per 100 patient-days, rather than DDD per 1,000 inhabitants, because we did not analyse hospital-wide antimicrobial use, but use in selected wards only.

Data for comparison of community antimicrobial use in Israel versus that in European countries, as determined by the European pooled-mean in 2012 and 2017, were taken from the 2018 summary published by the European Surveillance of Antimicrobial Consumption Network (ESAC-Net), a network managed and coordinated by the European Centre for Disease Prevention and Control [1]. To facilitate comparison with Israeli data, which includes individual ATC categories reported by ESAC-Net as ‘other’, data were compiled both as categorised by ESAC-Net and as categorised by the NCIC.

### Statistics

Linear regression was performed to analyse time trends in antimicrobial use with p ≤ 0.05 defined as statistically significant. All calculations were performed with VassarStats (Vassar College, Poughkeepsie, New York, United States).

### Ethical statement

Approval by an ethical committee was unnecessary, as this was a non-interventional study evaluating anonymised data that were collected for public health purposes and are publically available on the Israeli MOH website.

## Results

Data were available from all HMOs for the entire period. Data from acute care hospitals were available for only a subset of hospitals in the first 2 years (15/28 in 2012 and 22/28 in 2013), but all 28 hospitals in Israel reported data thereafter. In 2017, one hospital reported data as antimicrobials consumed by patients and not as pharmacy dispensing and because of this discrepancy, data from that hospital were not included in the analysis for that year. Antimicrobial use data from the PACHs were initially not available for all institutions (6/15 reported in 2012 and 13/15 reported in 2014), but as of 2016, all 15 PACHs reported.

**Figure 1:**
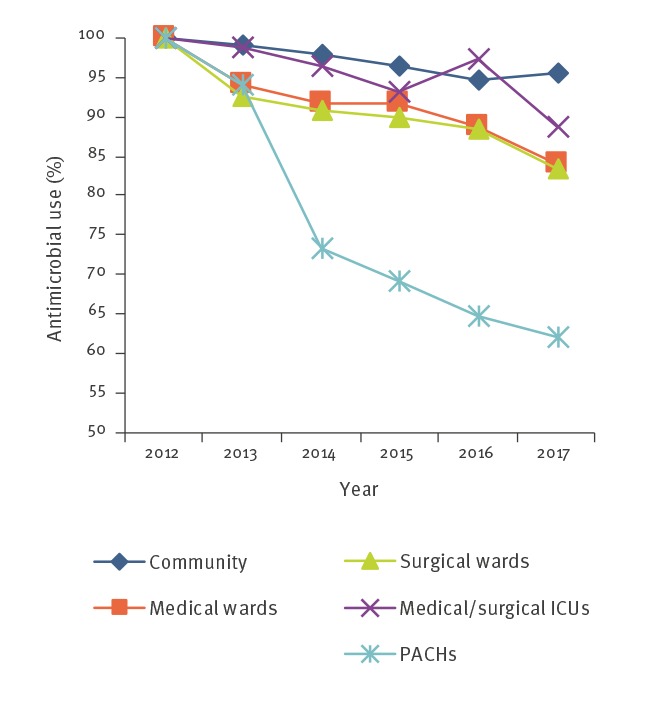
Antimicrobial use by patient population, Israel, 2012–2017

ICUs: intensive care units; PACHs: post-acute care hospitals.

Between 2012 and 2017 there was a statistically significant decrease in antimicrobial use in each type of healthcare institution studied (Figure 1).

### Community

**Figure 2:**
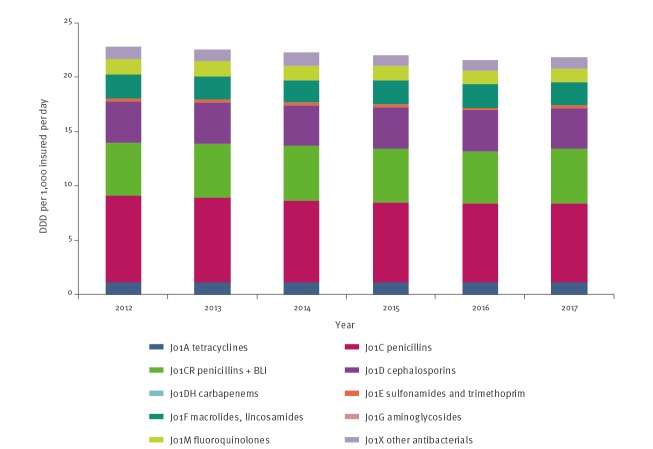
Antimicrobial use in the community by antibiotic category, Israel, 2012–2017

BLI: Beta-lactamase inhibitors; DDD: defined daily doses.

**Figure 3:**
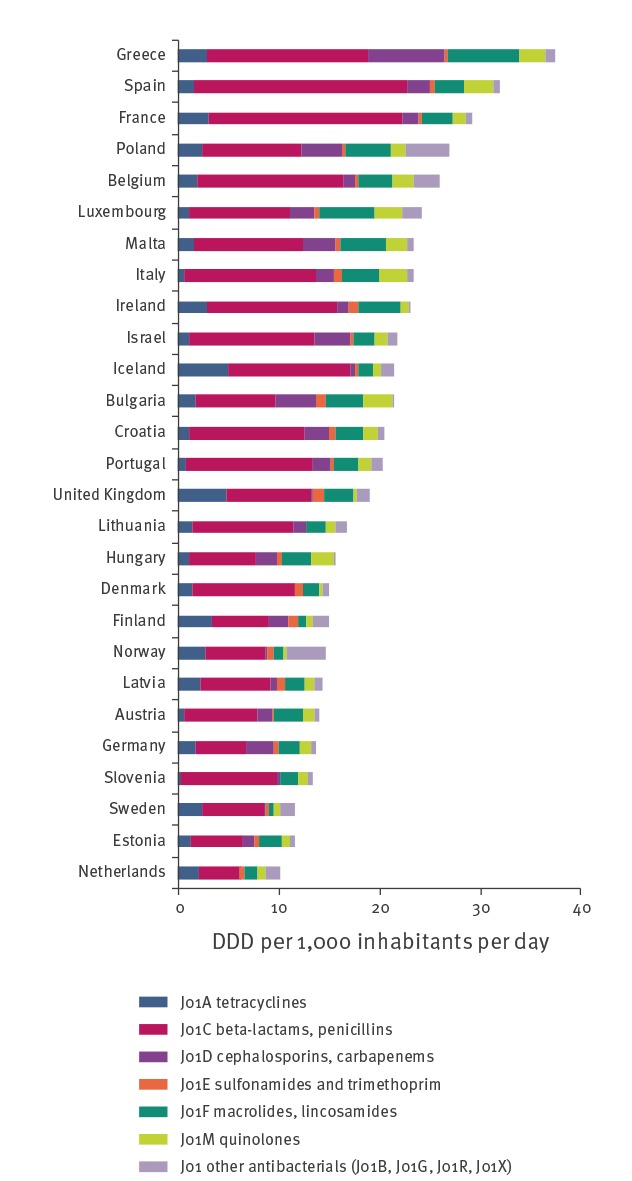
Antimicrobial use in the community, comparison with ESAC-Net 2017 data, Israel, 2012–2017

In the community, the total DDD per 1,000 insured per day declined 4.4%, from 22.8 in 2012 to 21.8 in 2017 (p = 0.004) (Table and Figure 2). Most of the decrease was because of significant declines in the use of penicillins (p = 0.033) and fluoroquinolones (p = 0.003). While three of the HMOs reported similar usage data during all years studied, the fourth reported consumption rates ca 20% higher than the others in 2012. It was this fourth HMO that saw the greatest decrease in antimicrobial use, accounting for most of the overall decrease in the community. Two of the HMOs did not report any difference in consumption during the period analysed. Compared with countries reporting to ESAC-Net, the rate of antimicrobial use in the community in Israel declined from 105.1% of the European pooled-mean in 2012 to 100.0% in 2017. In 2017, the rate of antimicrobial use in the community in Israel fell within the middle third of countries reporting to ESAC-Net (Figure 3).

**Table:** Antimicrobial use by patient population and antimicrobial category, Israel, 2012–2017

Antimicrobial category	Antimicrobial use						p value
	2012	2013	2014	2015	2016	2017	
**Community (DDD/1,000 insured/day)**							
J01A	1.1	1.2	1.1	1.1	1.1	1.1	0.441
J01C	12.9	12.7	12.6	12.3	12.1	12.4	0.033
J01D	3.8	3.8	3.7	3.8	3.8	3.7	0.414
J01E	0.2	0.2	0.2	0.2	0.2	0.3	0.158
J01F	2.2	2.1	2.0	2.3	2.2	2.1	0.924
J01M	1.4	1.4	1.3	1.3	1.2	1.2	0.003
All other J01 classes	1.1	1.2	1.2	1.0	1.0	1.0	0.116
**Total**	**22.8**	**22.6**	**22.3**	**22.0**	**21.6**	**21.8**	**0.004**
**Medical/surgical intensive care units (DDD/100 patient-days)**							
J01A	3.6	3.0	2.7	3.3	2.9	3.3	0.709
J01C	39.1	35.8	36.6	34.2	35.0	31.8	0.014
J01D	41.6	41.3	38.8	39.7	41.8	39.8	0.582
J01E	3.0	2.4	4.1	3.8	3.7	2.7	0.754
J01F	12.3	11.1	9.9	10.1	10.8	10.8	0.297
J01M	15.0	13.7	13.7	14.0	14.8	11.5	0.213
All other J01 classes	39.8	45.2	43.0	38.9	41.1	37.3	0.270
**Total**	**154.4**	**152.5**	**148.8**	**144.0**	**150.1**	**137.2**	**0.041**
**General surgical wards (DDD/100 patient-days)**							
J01A	1.0	0.7	0.6	0.5	0.5	0.5	0.024
J01C	41.9	37.9	35.6	34.6	34.7	32.6	0.007
J01D	22.1	24.1	24.3	25.1	24.8	26.1	0.010
J01E	0.4	0.7	0.4	0.6	0.5	0.5	0.932
J01F	4.5	3.8	3.8	3.8	3.6	3.4	0.021
J01M	20.2	15.2	16.7	15.6	15.2	12.1	0.036
All other J01 classes	22.7	22.1	20.5	20.8	20.0	18.9	0.002
**Total**	**112.8**	**104.5**	**102.3**	**101.3**	**99.6**	**94.2**	**0.004**
**General medical wards (DDD/100 patient-days)**							
J01A	5.4	5.2	4.9	5.1	5.0	4.8	0.036
J01C	29.1	26.4	26.1	26.0	26.0	23.6	0.021
J01D	30.4	29.0	28.3	28.5	28.0	27.4	0.008
J01E	1.3	1.3	1.4	1.3	1.1	1.2	0.188
J01F	11.2	10.3	10.5	10.8	10.1	9.8	0.068
J01M	14.8	13.6	12.2	11.5	10.3	9.0	< 0.001
All other J01 classes	7.8	8.3	8.2	8.5	8.2	8.3	0.231
**Total**	**100.0**	**94.1**	**91.7**	**91.7**	**88.7**	**84.0**	**0.002**
**Post-acute care hospitals (DDD/100 patient-days)**							
J01A	0.5	0.2	0.3	0.4	0.3	0.4	0.924
J01C	9.3	8.9	6.3	5.7	5.9	5.7	0.020
J01D	8.4	6.5	5.4	5.4	4.9	4.9	0.020
J01E	0.8	1.0	0.7	0.8	0.7	0.7	0.213
J01F	1.3	1.1	1.0	0.9	0.8	0.8	0.002
J01M	5.1	4.6	3.8	3.5	3.0	2.8	< 0.001
All other J01 classes	3.5	5.1	3.8	3.4	3.0	2.8	0.148
**Total**	**29.1**	**27.4**	**21.3**	**20.1**	**18.8**	**18.1**	**0.005**

DDD: defined daily doses.

DDD: defined daily doses.

This figure is adapted from the European Centre for Disease Prevention and Control’s report, Antimicrobial consumption [1].

### Acute care hospitals

**Figure 4:**
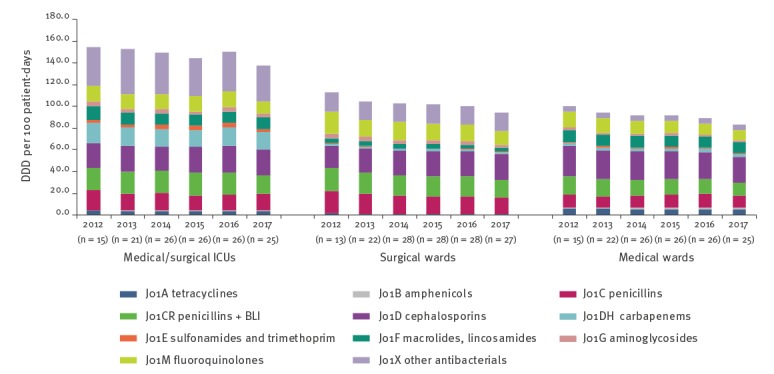
Antimicrobial use in acute care hospitals by antimicrobial category and ward, Israel, 2012–2017

BLI: Beta-lactamase inhibitors; DDD: defined daily doses; ICUs: intensive care units.

Among acute care hospitals, antimicrobial use decreased between 2012 and 2017 (Table and Figure 4). This decrease was significant in all three ward types.

The number of acute care hospital wards reporting that year is shown under the year.

Within medical wards, the combined DDD per 100 patient days was 100.0 in 2012, declining 16.0% to 84.0 by 2017 (p = 0.002). Significant declines in antimicrobial use were seen for fluoroquinolones (14.8 to 9.0 DDD/100 patient-days, p < 0.001) and beta-lactams, specifically beta-lactam/beta-lactamase inhibitors (16.9 to 11.7 DDD/100 patient-days, p < 0.001) and cephalosporins (27.8 to 24.9 DDD/100 patient days, p = 0.012). A significant increase was observed in the consumption of chloramphenicol (approved for use in Israel), from 0.8 to 1.6 DDD per 100 patient-days (p = 0.003).

In surgical wards, the rate dropped 16.5%, from 112.8 DDD per 100 patient-days in 2012 to 94.2 in 2017 (p = 0.004). Significant declines were seen for most antimicrobial groups, with the greatest absolute decreases observed for fluoroquinolones (20.2 to 12.1 DDD/100 patient-days, p = 0.036), penicillins (20.2 to 15.9 DDD/100 patient-days, p = 0.022) and beta-lactam/beta-lactamase inhibitors (21.7 to 16.7 DDD/100 patient-days, p = 0.003). A significant increase was observed in the consumption of cephalosporins, from 20.5 to 24.1 DDD per 100 patient days (p = 0.01).

In medical/surgical ICUs, antimicrobial use dropped 11.1%, from 154.4 DDD per 100 patient days in 2012 to 137.2 in 2017 (p = 0.041). While consumption in most antimicrobial categories decreased, no decline reached statistical significance.

### Post-acute care hospitals

**Figure 5:**
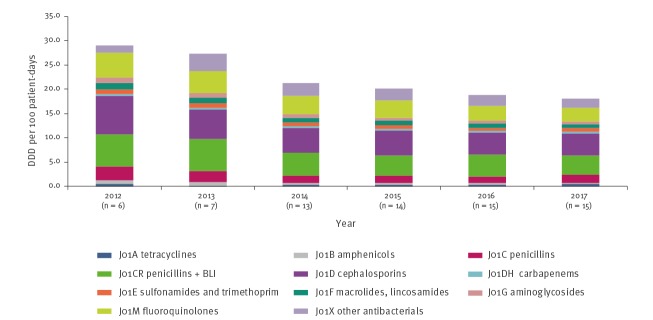
Antimicrobial use in post-acute care hospitals by antimicrobial category, Israel, 2012–2017

BLI: Beta-lactamase inhibitors; DDD: defined daily doses.

PACHs demonstrated a 37.8% decline in antimicrobial use during the reporting period, the largest for all three institutional types, from 29.1 DDD per 100 patient days in 2012 to 18.1 in 2017 (p = 0.005, Table and Figure 5). This decline was the result of a significant decrease in the use of most antimicrobial groups, with the greatest declines observed for cephalosporins (8.0 to 4.4 DDD/100 patient-days, p = 0.016), beta-lactam/beta-lactamase inhibitors (6.6 to 4.1 DDD/100 patient-days, p = 0.013) and fluoroquinolones (5.1 to 2.8 DDD/100 patient-days, p < 0.001).

The number of post-acute care hospitals reporting that year is shown under the year.

## Discussion

Following the 2012 launch by the Israeli NCIC of a nationwide antimicrobial stewardship intervention [18], a decrease in antimicrobial use was observed in Israel in the community, in medical and surgical wards as well as medical/surgical ICUs within acute care hospitals, and in PACHs. Most of this observed decrease was attributable to declines in use of fluoroquinolones and penicillins, including beta-lactam/beta-lactamase inhibitors.

Although the decrease in antimicrobial use was seen almost universally across the healthcare spectrum in Israel, considerable variation existed between different institutions. In the community, differences were observed between HMOs, with the bulk of the overall decrease in antimicrobial use occurring in the HMO that had the greatest initial rate of consumption. In the acute care hospital setting, although significant decreases were seen in consumption rates of all ward types, inter-hospital variability was significant. There were numerous wards and medical/surgical ICUs where no change was seen over time and others in which antimicrobial use increased. In contrast, a trend towards decreased antimicrobial use was nearly uniform among PACHs, with only a few reporting increased or static rates of consumption.

Israel is a country with relatively high rates of AMR, comparable to some southern Europe countries [21]. Previous nationwide antimicrobial stewardship programs that have been successful, e.g. in Sweden, France and Scotland [22-24], were implemented in countries where the baseline rates of AMR and antimicrobial use were lower than those in Israel. It is likely that as overall antimicrobial use decreases, it becomes ever more challenging to reduce it further and this may account for the relatively static rates observed in many countries with below-average antimicrobial use [1]. As noted above, the significant decreases in antimicrobial use that we observed are in contrast to a lack of change in antimicrobial use in these settings across the majority of European countries reporting to ESAC-Net within the same time period, particularly in countries with rates of consumption greater than the population-weighted mean [1]. However, the degree of reduction within Israel was uneven, with considerably greater drops in use in inpatient settings than in the community, findings that will inform future stewardship interventions.

This report has a number of limitations. As noted, our data relied on antimicrobial dispensing records. Discrepancies can arise between quantities dispensed and consumed, for example when a patient fails to complete a course of therapy or when the decision is made to change antimicrobials mid-way through treatment. In both cases, the quantity of antimicrobial consumed will be less than that dispensed. Thus, our data may overestimate rates of antimicrobial use; however, it is not anticipated that this problem affects the analysis of temporal trends. As data reported to ESAC-Net may suffer from a similar issue given that rates of antimicrobial use are based on national sales or reimbursement data [25], the issue should also not affect the comparison with European data. A second limitation is the lack of a comparator for our data from acute care hospitals and PACHs because of the use of a different denominator. Data on antimicrobial use in hospitals are reported in ESAC-Net as DDD per 1,000 inhabitants per day rather than per 100 patient-days. Data from the Global Point Prevalence Survey reflect only the percentage of patients receiving antimicrobials on the survey date [26]. The decision to gather ward-level rather than hospital-level data in acute care hospitals in Israel was pragmatic, permitting timely feedback to units where changes in antimicrobial use can be relatively easily implemented. Furthermore, it enabled the identification of departments whose antimicrobial use deviates significantly from the mean, assisting antimicrobial stewardship efforts.

In conclusion, following the introduction of a nationwide antimicrobial stewardship intervention, there was an observed decrease in the rates of antimicrobial use in all types of studied healthcare institutions in Israel between the years 2012 and 2017.
